# Supplementary figures and images for: The respiratory cycle modulates distinct dynamics of affective and perceptual decision-making
Source: PLoS Comput Biol. 2025 May 27;21(5):e1013086. doi: 10.1371/journal.pcbi.1013086 (PMC12240353; doi:10.1371/journal.pcbi.1013086)

**S1 Figure. T-tests.**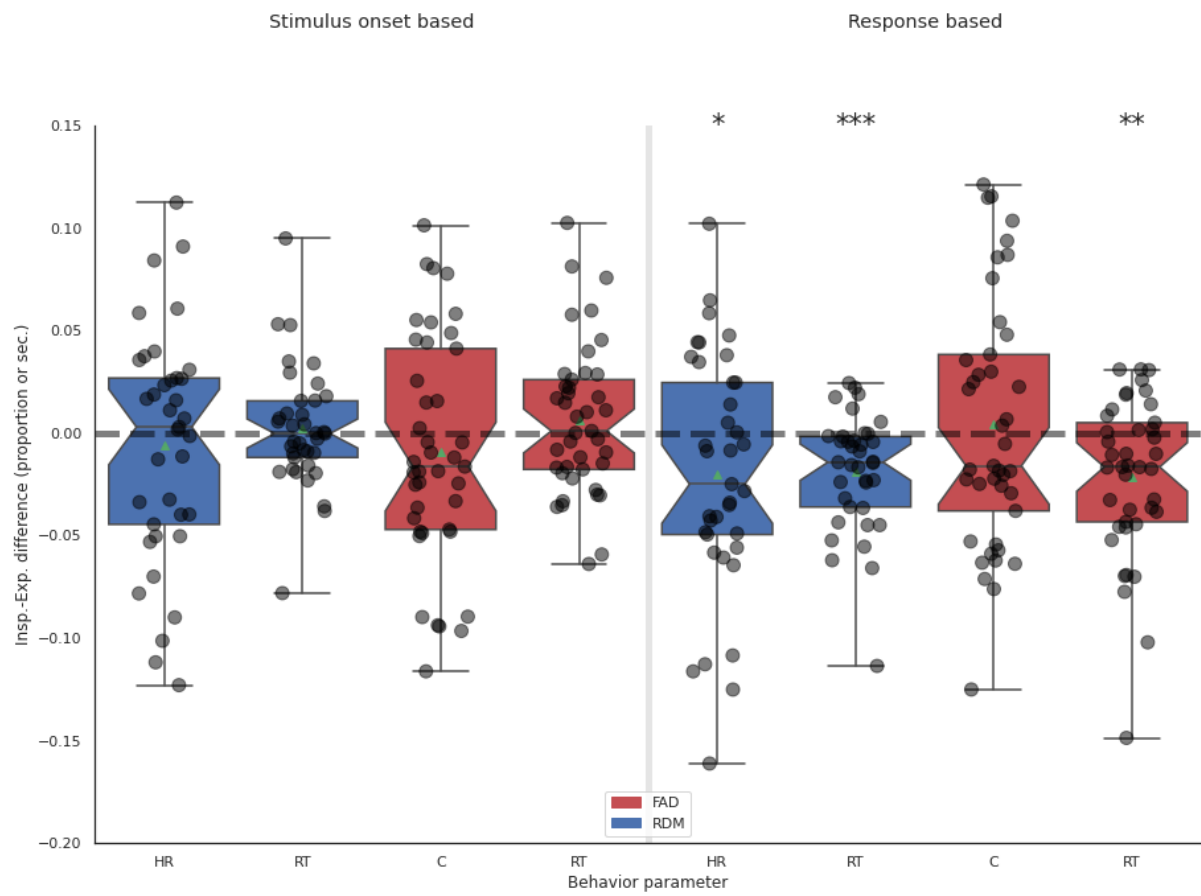

Supplement: S1 Fig — Difference in task behaviour during inspiration vs expiration per participant. Positive values indicate higher values during inspiration compared to expiration. HR: Hit rate, RT: Reaction time, C: choice (proportion ‘happy’-responses). Y-axis show difference in proportions for HR and C and difference in median RT for RT. Notches indicate 95%-CI of the median. RDM: Random dot motion, FAD: Face Affect Discrimination. *p < 0.05, **p < 0.01, ***p < 0.001. (PDF) [file pcbi.1013086.s001.pdf]

**S2 Figure. Sampling traces, RDM model.**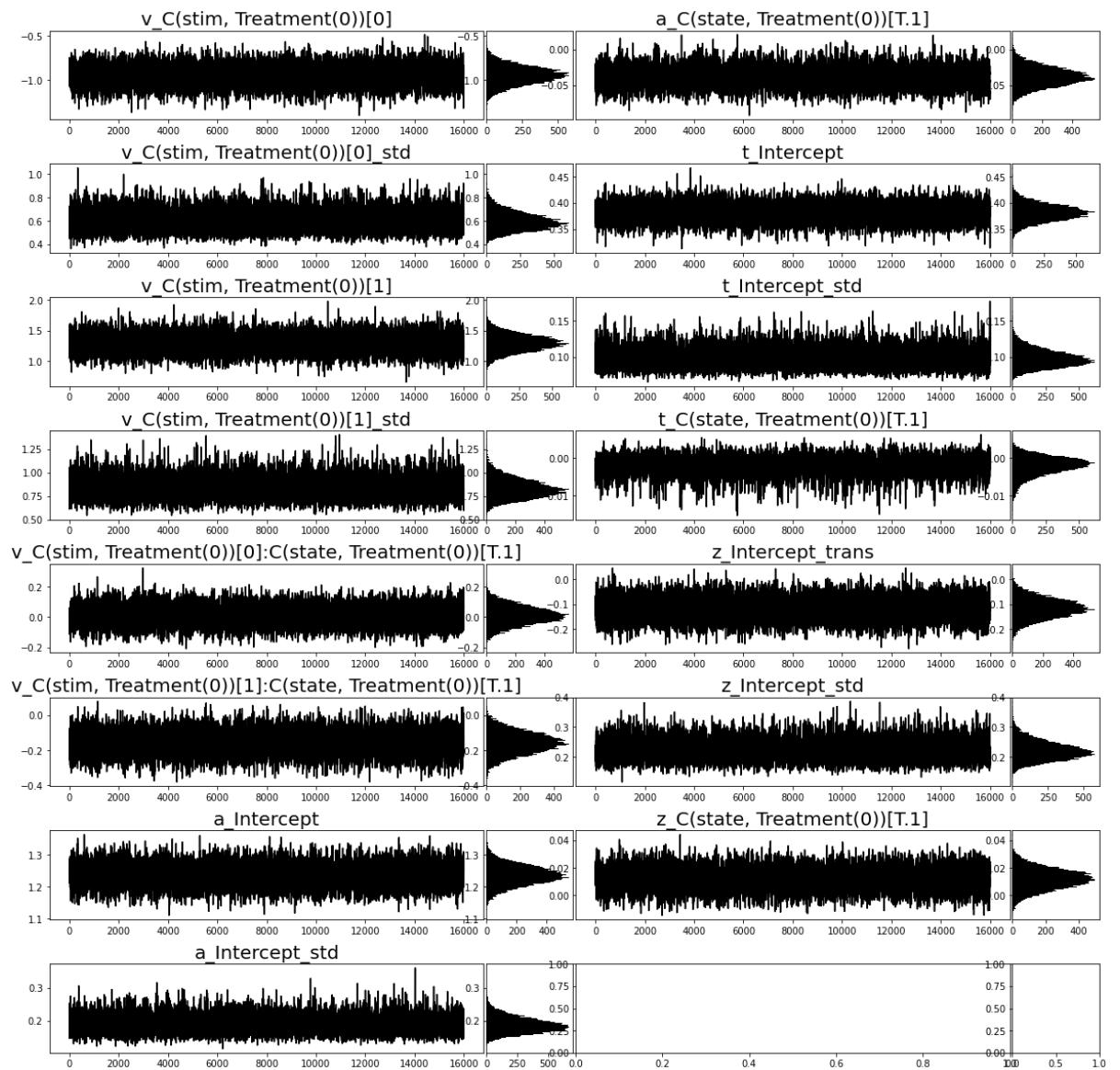

Supplement: S2 Fig — Caterpillar plots showing sampling traces for the Random Dot Motion model for all group level parameters. (PDF) [file pcbi.1013086.s002.pdf]

**S3 Figure. Sampling traces, FAD model.**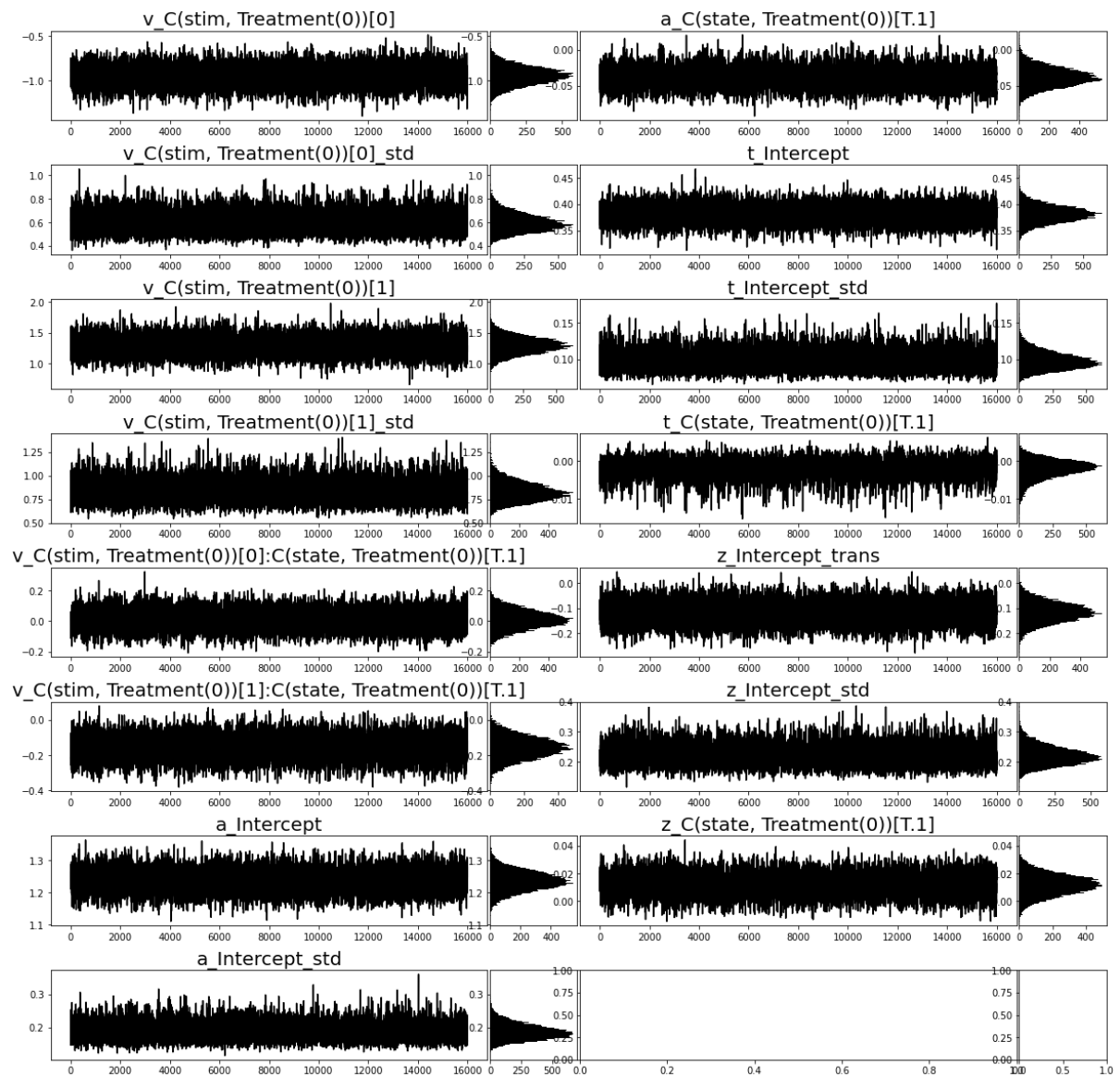

Supplement: S3 Fig — Caterpillar plots showing sampling traces for the Face Affect Discrimination model for all group level parameters. (PDF) [file pcbi.1013086.s003.pdf]

**S4 Figure. Face morphing.**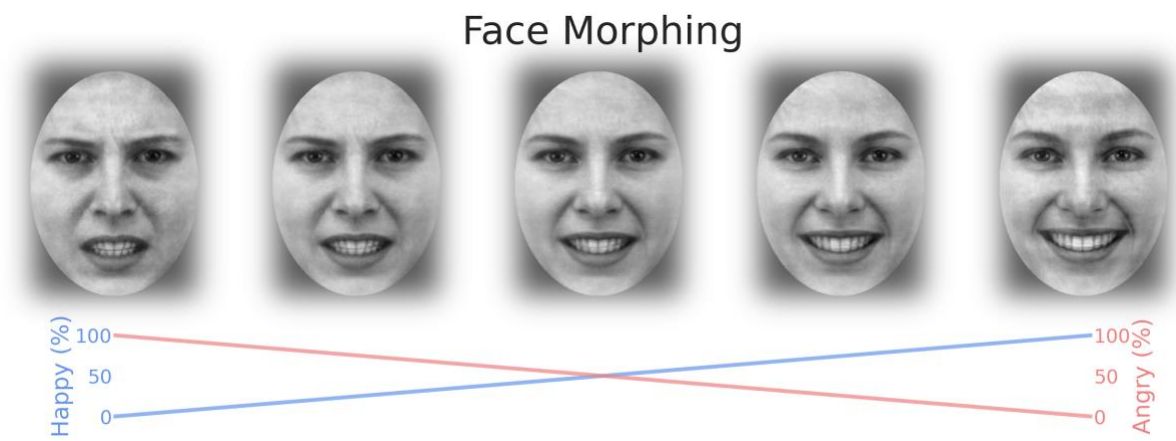

Supplement: S4 Fig — Angry and happy face stimuli were morphed to create 201 categorical stimulus levels ranging from 100% angry and 0% happy to 100% happy and 0% angry. (PDF) [file pcbi.1013086.s004.pdf]

**S5 Figure. Histograms of event distribution over continuous (circular) respiratory phase.**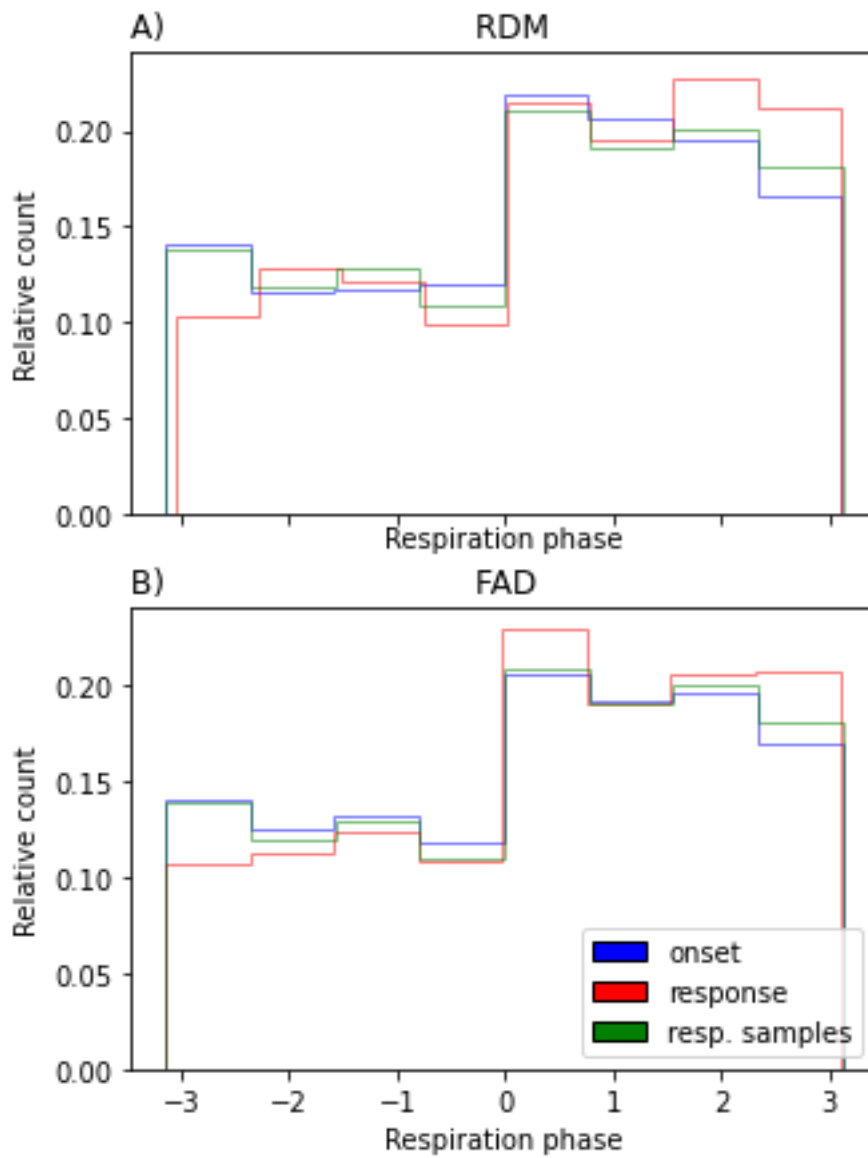

Supplement: S5 Fig — A) Random dot motion, B) Face affect discrimination. Histograms showing the relative frequency of stimulus onsets (blue lines) and responses (red lines) over the respiratory cycle. Negative pi to zero denotes the inspiratory phase and zero to positive pi denotes the expiratory phase. The green line represents the time spent in each of the phase bins. We note two things 1: As expected more time is spent at the expiratory phase compared to the inspiratory phase. 2: Relative frequency of stimulus onsets and responses follow the time spent in each phase bin. (PDF) [file pcbi.1013086.s005.pdf]

**S7 Figure. Task performance over the respiratory cycle.**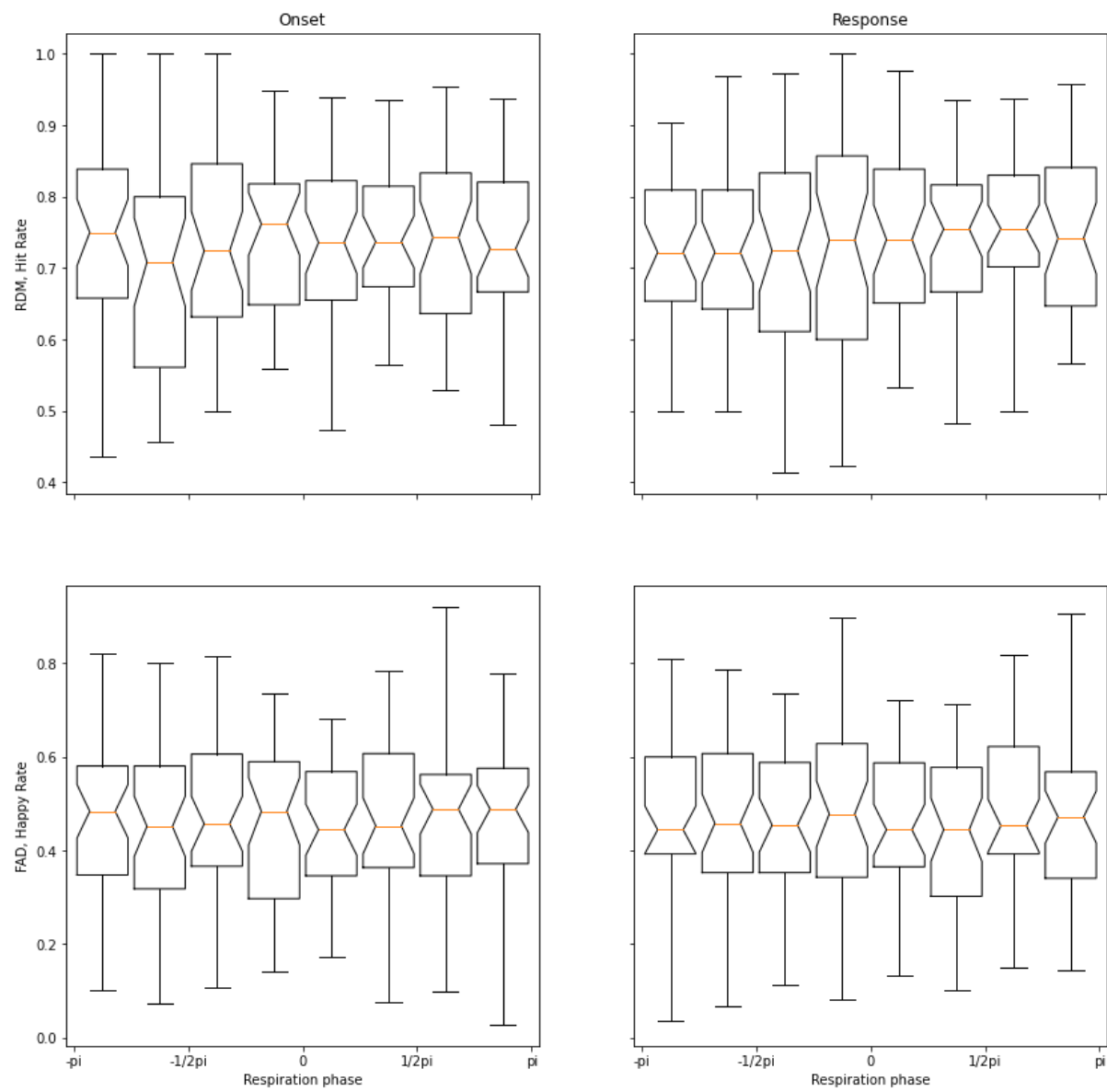

Supplement: S7 Fig — Boxplots representing behaviour in eight discrete respiratory phase bins. Stimulus- and response-locked hit rate for RDM and happy rate for FAD. (PDF) [file pcbi.1013086.s007.pdf]
