# Supplementary material for: The respiratory cycle modulates distinct dynamics of affective and perceptual decision-making
Source: PLoS Comput Biol. 2025 May 27;21(5):e1013086. doi: 10.1371/journal.pcbi.1013086 (PMC12240353; doi:10.1371/journal.pcbi.1013086)

S6 Figure. Box Plots representing distribution of hit rates per breath duration quartile bin over subjects for RDM.

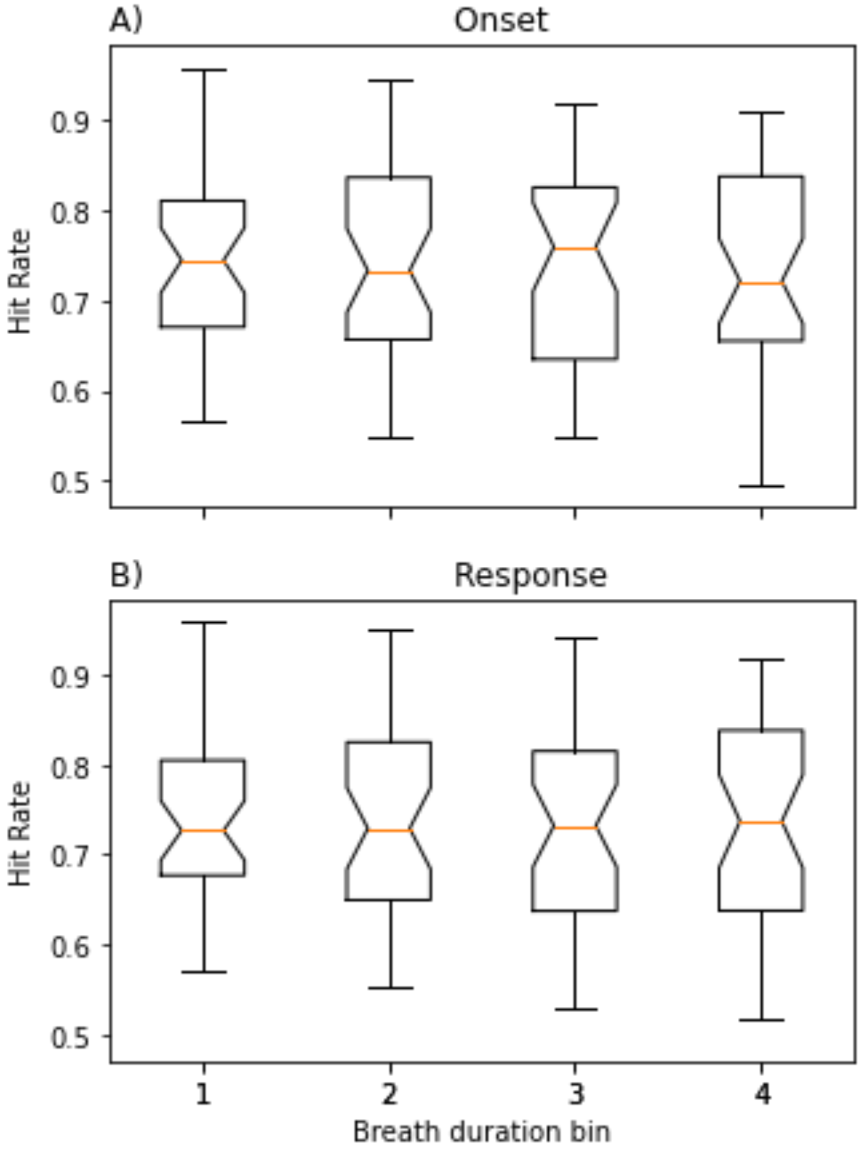

Supplement: S6 Fig — A) Stimulus-locked, B) Response-locked. (PDF) [file pcbi.1013086.s006.pdf]
