# Supplementary material for: The respiratory cycle modulates distinct dynamics of affective and perceptual decision-making
Source: PLoS Comput Biol. 2025 May 27;21(5):e1013086. doi: 10.1371/journal.pcbi.1013086 (PMC12240353; doi:10.1371/journal.pcbi.1013086)
Supplement: S1 Table — Statistical analysis of behaviour during inspiration vs expiration. Diff. and 95%-CI refers to the mean difference between inspiration and expiration measured in proportions for HR and C and in seconds for RT. Positive values indicate higher values during inspiration compared to expiration. T and p-values refers to paired t-tests. BF10 and BF01 is the Bayes factor in favour of the alternative and the null hypothesis respectively. Behav.: Behavioural parameter. HR: Hit rate, RT: Reaction time, C: choice (proportion ‘happy’-responses), RDM: Random dot motion, FAD: Face Affect Discrimination. (PDF) [file pcbi.1013086.s008.pdf]

S1 Table. T-tests.

Grouping	Task	Behav.	Diff.	95%-CI	T	p-val.	BF10	BF01
Onset	RDM	HR	-0.0059	[-0.02-0.01]	-0.6379	0.5276	0.2	4.673
	RDM	RT	0.0021	[-0.01-0.01]	0.4262	0.6725	0.2	5.208
	FAD	C	-0.0093	[-0.03-0.01]	-1.0815	0.286	0.3	3.436
	FAD	RT	0.0062	[-0.01-0.02]	1.0926	0.2811	0.3	3.401
Response	RDM	HR	-0.0202	-0.04--0.0]	-2.0885	0.0439	1.2	0.816
	RDM	RT	-0.0189	[-0.03--0.01]	-4.0651	0.0002	108.4	0.009
	FAD	C	0.004	[-0.02-0.02]	0.4184	0.6779	0.2	5.464
	FAD	RT	-0.0212	[-0.03--0.01]	-3.533	0.0011	28.9	0.035
