# Supplementary material for: The respiratory cycle modulates distinct dynamics of affective and perceptual decision-making
Source: PLoS Comput Biol. 2025 May 27;21(5):e1013086. doi: 10.1371/journal.pcbi.1013086 (PMC12240353; doi:10.1371/journal.pcbi.1013086)
Supplement: S2 Table — For each task and trial grouping mean ± SD in percentage points of excluded trials per step relative to the presented 320. Behav.: Exclusion based on behaviour, resp.: respiratory, RDM: Random dot motion, FAD: Face Affect Discrimination. (PDF) [file pcbi.1013086.s009.pdf]

S2 Table. Trial exclusion.

Exclusion	RDM		FAD	
Behav.	0.4, 0.6		0.7, 0.8	
	Onset locked	Response locked	Onset locked	Response locked
Manual bad resp. signal	1.7 ± 2.6	1.7 ± 2.6	2.1 ± 3.0	2.1 ± 3.0
Trial on peak/trough	5.4 ± 1.3	5.3 ± 1.7	5.0 ± 1.5	5.2 ± 1.5
Total	7.6 ± 2.6	7.4 ± 3.0	7.9 ± 3.3	8.0 ± 3.5
