# Supplementary material for: The respiratory cycle modulates distinct dynamics of affective and perceptual decision-making
Source: PLoS Comput Biol. 2025 May 27;21(5):e1013086. doi: 10.1371/journal.pcbi.1013086 (PMC12240353; doi:10.1371/journal.pcbi.1013086)
Supplement: S3 Table — Reaction times (ms) following trial exclusion steps. For each task and trial grouping mean ± SD reaction times over responses for all subjects are presented. Behav.: Exclusion based on behaviour, resp.: respiratory. (PDF) [file pcbi.1013086.s010.pdf]

S3 Table. Trial exclusion, reaction times.

Exclusion	RDM		FAD	
No exclusion	693 ± 274		756 ± 305	
Bad subjects	688 ± 273		-	
Behav.	688 ± 273		756 ± 305	
	Onset locked	Response locked	Onset locked	Response locked
Manual bad resp. signal	0.687 ± 0.273	0.687 ± 0.273	0.754 ± 0.305	0.754 ± 0.305
Trial on peak/trough	0.688 ± 0.274	0.688 ± 0.275	0.755± 0.305	0.754 ± 0.305
