# Supplementary material for: The respiratory cycle modulates distinct dynamics of affective and perceptual decision-making
Source: PLoS Comput Biol. 2025 May 27;21(5):e1013086. doi: 10.1371/journal.pcbi.1013086 (PMC12240353; doi:10.1371/journal.pcbi.1013086)
Supplement: Attachment — Submitted filename: Reviews_letter.pdf [file pcbi.1013086.s012.pdf]

We thank all three reviewers for their insightful comments and provide a point-by-point response to their suggestions below. In response to the reviewers' suggestions, we have:

1. Clarification of Task Selection: We expanded the introduction and methods to explain our choice of the motion discrimination and affect discrimination tasks, highlighting how they allow us to investigate respiratory modulation across basic sensory processing and emotional interpretation.

2. Integration with Interoception Literature: We discussed the two branches of interoception research—perceptual and affective stimuli—and situated our study within this framework, addressing how it contributes to understanding disparities in previous findings.

3. Consideration of Potential Confounds: We acknowledged the potential influence of pupillary dynamics and eye movements, adding discussion on how future research could incorporate multimodal recordings to deepen understanding.

4. Sample Size Justification: We provided justification for our sample size and trial numbers, demonstrating that our study offers increased statistical power compared to prior studies.

5. Clarification of Analytical Approach: We explained why an ANOVA design was not suitable due to design constraints and potential violations of statistical assumptions, focusing instead on our computational modeling approach.

6. Analysis of Breathing Patterns: We analyzed whether participants altered their breathing patterns in relation to task performance, finding no significant modulation, and included these results in the manuscript.

7. Addressing RT and Accuracy Dependency: We clarified that our modeling approach accounts for the dependency between reaction times and choices, ensuring the validity of our interpretations.

8. Editorial Enhancements: We improved figure legibility, updated terminology for clarity, enhanced figure captions, and added relevant statistics as per the reviewers' suggestions.

In what follows, the reviewers' comments are presented in gray, our responses in blue, changes made during the revision in red.

Reviewer #1:
The manuscript by Brændholt et al reports the co-modulation of decision-making dynamics with respiration cycle during two different discrimination tasks, one based on motion coherence and another on affect in a face. Utilizing a computational model, the paper attempts at yielding mechanistic insights and an explanation for the observed behavioral effects. The study tackles an interesting scientific issue and the authors utilize pertinent computational methods for modeling the behavioral data. The paper is nicely written and structured with a clear message. In order to further contextualize the presented results, I add some aspects which the authors may consider. These may help in shedding further light on the presented findings.

Please find suggestions/comments below:

- It is unclear if the subjects were instructed to fixate during the presentation of the motion discrimination stimuli and if their eye movements were monitored to manage their oculomotor behavior. This is important, as vertically drifting motion stimuli utilized here may elicit eye movements in the subjects. Further, it has been previously suggested that the eye movements, such as the optokinetic nystagmus elicited in response to vertical motion may be asymmetric with different gains for upwards or downward direction (see, for e.g. – van den Berg and Collewjin, Experimental brain Research, 1988, https://pubmed.ncbi.nlm.nih.gov/3384058/). It is of note that the manuscript mentions an asymmetric reduction in drift rate for motion stimuli (Line 169- 171). A characterization of the eye movement dynamics in the experiments reported in the manuscript may help ascertain if they display an asymmetric nature. Further, the modeling results may be interpreted in the light of the findings related to the eye movement dynamics.

Unfortunately we did not collect any eye tracking data. However, we agree that considerations of potential eye movements are an important step forward in understanding respiratory modulation of behavior. Here, we attempted to control for or at least limit the possibility of such effects through a few key aspects of our experimental design. In particular, we utilized a head and chin rest device to stabilize the position of the head throughout the experiment, which to our knowledge, has not been utilized in previous similar experiments. We also instructed participants to fixate on the central point, which was clearly marked by a fixation dot. Finally, we chose very brief stimulus presentation durations (e.g., 400 ms), as these have been shown to reduce the possibility and influence of eye movements.

Nevertheless, we acknowledge that even in the face of these improvements, it is possible

that some degree of respiratory effects may be driven by oculomotor responses. Actually, it is a bit more complicated than that as Galvez-Pol et al have shown that respiratory rhythms appear to also drive saccades to some degree. To acknowledge this complexity, we now introduce the following limitation to the discussion:

“More generally, understanding the respiratory modulation of the brain and behavior ultimately requires a multi-modal and multi-organ perspective [See for example, 42–44]. In this study, we utilized computational modeling to explain how breathing rhythms influence choice, accuracy, and decision speed, extending and replicating previous findings. However, brain-body interactions encompass a complex array of physiological processes, including various neurotransmitters such as noradrenaline and autonomic signals from the heart, gut, and other visceral systems. This complexity highlights a limitation of our current work and suggests directions for future research. Future studies could employ multimodal recordings — such as pupillometry, electrocardiograms, and electrogastrography — alongside respiratory measurements, thereby expanding computational models to account for both uni- and multimodal brain-body interactions. Indeed, recent findings on eye movements and pupillary dynamics [45], as well as animal studies demonstrating that interoceptive vagal pathways involve both multimodal and unimodal afferents [46], indicate that a comprehensive understanding of respiratory-brain coupling necessitates a multimodal approach.“

(p. 18-19)

- Recent work suggests a respiratory pupillary phase effect (Schaefer et al, 2024, https://www.biorxiv.org/content/10.1101/2024.06.27.599713v1), that is - fluctuations in pupil diameter co-occur with inhalation and exhalation. In particular, the study found that the pupil size is smallest around inhalation onset and largest around peak exhalation. In addition, pupillary dynamics have been reported to be associated with performance during a discrimination task (e.g. Mathot and Ivanon, 2019) and also associated with other cognitive processes (Ebitz ad Moore, 2018, https://www.ncbi.nlm.nih.gov/pmc/articles/PMC6350273/). Therefore, the authors may consider quantifying modulation of pupillary dynamics in conjunction with respiration as subjects perform the motion discrimination or affect discrimination task. This may help characterize, if the observed effects are associated with changes in the pupil diameter, which may help understand the association of respiratory cycle with the decision making dynamics better.

We appreciate this insightful suggestion, which aligns well with your previous point. We fully agree that future work should incorporate multimodal peripheral recordings, including pupillary signals, to gain a more comprehensive understanding of higher-order interactions. However, the focus of our current analysis was specifically on whether

different respiratory states (inspiration vs. expiration) corresponded to changes in computational parameters within our decision-making model.

The reviewer raises an important point regarding the potential influence of arousal states, which can be inferred from pupillary dynamics. Given the known anatomical connections between the locus coeruleus (LC) and respiratory pacemaker nuclei in the brainstem, we recently explored the relationship between respiration and pupil dynamics (Kluger et al., Journal of Neuroscience, 2024). In this study, we found cross-frequency coupling between respiratory and pupillary rhythms (specifically in the hippus frequency) across three datasets. However, this coupling was significantly reduced during perceptual decision-making tasks compared to resting-state recordings. Additionally, directed functional connectivity analyses indicated that this coupling was driven by respiration modulating pupil dynamics, not the other way around.

While we did not specifically control for pupil diameter dynamics in the current study, our findings suggest that it is unlikely that pupillary modulations account for the behavioral effects observed in our modeling. Rather, respiration likely serves as the primary driver of both behavioral changes and arousal markers, such as pupil diameter. We have added this point to the new limitation section of the discussion:

“Note, however, that both unidirectional anatomical connections from the preBötzinger complex to LC [30] as well as recent findings from directed functional connectivity analyses [45] suggest respiration as the primary driver of both behavioral changes and arousal markers.”

(p. 19)

- The manuscript reports that the stimuli utilized for the discrimination tasks were titrated for each individual. Therefore, one may wonder if the observed effects only occur under such constrained stimulus conditions. The authors may consider discussing this point. In general, would similar co-modulation of respiratory and decision dynamics be also observed if such a stimulus threshold is not chosen.

We chose this approach to avoid ceiling and floor effects and to ensure that respiratory effects could be observed without stimulus-specific biases. However, we acknowledge that this raises an important question about whether the observed effects would also occur under less constrained stimulus conditions. While other studies have found co-modulation of respiratory and decision dynamics using un-thresholded stimuli, the tasks and types of co-modulation differed from our study. This highlights the necessity of discussing this point further. To address this, we have expanded the discussion in our manuscript to consider the potential limitations of our approach and how future research might explore the generalizability of these effects:

"Our study has specific limitations that could be addressed in future investigations. Participants viewed stimuli presented at peri-threshold levels to control for stimulus-specific biases and avoid ceiling or floor effects. However, this manipulation could introduce confounds related to cognitive fatigue or performance drift over time. Although our inspection of performance across trial blocks highlighted a high relative stability, future studies could utilize adaptive thresholding methods. This would not only mitigate potential performance drift but also allow for the estimation of respiratory-modulated psychophysical curves. For instance, a previous study [17] employed adaptive thresholding to re-estimate psychophysical curves across different respiratory phases, revealing that perceptual thresholds were modulated by breathing—an effect linked to neural excitability changes. Combining our computational modeling approach with adaptive psychophysics could provide deeper insights into how respiratory-driven excitability fluctuations influence perceptual evidence accumulation, an area where we observed only weak effects in the present study."

(p. 17)

- A general point is related to the interpretation that the respiration cycle - modulates - the dynamics of decision making. Such interpretation and similar statements in the text may suggest a causality to the reader between the two variables. Is it plausible that a co-occurring third factor, e.g. pupil diameter, or oculomotor behavior, etc may contribute to the observed reaction time related effects. Therefore, an analysis of oculomotor behavior and pupil diameter (as suggested earlier) would be helpful to add further context to the presented results. The authors may consider discussing these points depending upon the results they get, which would help to balance the overall message presented in the manuscript.

As we answered above, we unfortunately did not record eye movements or pupillometry. We agree that these analyses would be interesting (see the discussion earlier of Kluger et al, Journal of Neuroscience 2024). It is worth noting, however, that the respiratory signal remains unique in that it can be controlled voluntarily. This ‘active’ role of respiration is also stressed by Reviewer 2 and further supported by unidirectional anatomical connections from the preBötzinger complex to the locus coeruleus. Taken together, anatomical as well as functional evidence strongly suggest a distinction between respiration as an active modulator and pupil dilation as a passive read-out signal reflective of brain-body states with respect to arousal changes. We now consider this important issue in the discussion as follows:

“Finally, while our study builds on numerous studies suggest a causal role for breathing in modulating the brain, future work could benefit from the application of respiratory manipulations to further elucidate the directionality of breathing on specific decision variables. “

(p. 19)

Minor comments:
Thank you, we have corrected these issues as suggested:

- The text on Supplementary figure 2 is not quite legible. Please consider revising it.

We have split figure Supplementary figure 2 into two new figures, Supplementary figure 2 and 3 and increased text size to increase legibility.

- In general, it may help to avoid the word ‘unique’ in the following lines - e.g. line 78, 244, 297, as this may suggest and be misunderstood as the ‘only’ mechanism.

All occurrences of the word ‘unique’ have been changed to ‘distinct’.

- Adding a description of the task within the caption of figure 1 may help the reader. At the same place, a description about the motion stimulus may be added – e.g. blue arrows denotes the coherent motion direction, while red arrows denote random directions, etc.

The caption of Figure 1 A has been updated to now read as follows:

“Fig 1: A) Trial structure of the random dot motion (RDM) and face affect discrimination (FAD) task. The task is to identify the coherent motion direction (blue arrows denote coherent motion, red arrows denote random motion. Arrows shown here for illustrative purposes) or decided whether face expressions are more happy or angry. [...]”

(p. 7)

- Perhaps it is mentioned in the manuscript and I missed this – please add the average reaction time (and the standard deviation) computed over all trials during the different discrimination paradigms before and after implementing the various exclusion criterion (reported in S5 table). Further, such a reaction time statistic may be added in the main text computed for trials grouped by respiratory state at stimulus onset and response.

We added a Table (S7 Table) presenting the consequences of trial exclusion on mean reaction time and standard deviation.

Finally, a section describing mean reaction times for onset and response based groping during inspiration and expiration has been added to the results section:

These results are in line with the descriptive statistics across subjects where RDM responses made during inspiration and expiration respectively had means of 675 ms ± 275, and 694 ms ± 274, just like response-locked FAD reaction times for inspiration and expiration was 737 ms ± 295, and 763 ± 309 respectively (±: SD). Stimulus-locked reaction times for inspiration and expiration respectively were 689 ms ± 273, and 688 ms ± 275, for RDM and 758 ms ± 306, and 753 ms ± for FAD.

(p. 9)

Reviewer #2:
The authors investigate the influence of respiratory rhythms on perceptual and affective processes beyond homeostatic survival. Recent studies in both humans and rodents suggest that neural and behavioral oscillations are modulated by the respiratory cycle. To examine these mechanisms, the authors conducted a psychophysical experiment with 41 participants who categorized dot motion and facial emotion stimuli in a standardized discrimination task. The findings revealed that inspiration accelerated responses in both domains. Using a hierarchical evidence accumulation model, it was determined that inspiration lowered evidential decision boundaries, leading participants to prioritize speed over accuracy in the motion task. Additionally, inspiration shifted the starting point of affective evidence accumulation, causing a bias towards more positive categorization of facial expressions. This study provides a novel computational understanding of how respiratory rhythms impact decision-making processes.

This is an interesting and timely manuscript. The paper significantly advances our understanding of the interplay between respiratory states and cognitive functions, it offers nice insights that could inform future studies in various fields. I recommend some minor changes, mostly to clarify concepts and better contextualize the study. Plus, I recommend another analysis: participants active coupling to the expected stimuli onset.

Introduction:

1. The authors should note why the choice of those physiological tasks (rationale for each individual task, and why both of them). Plus, note here or in the discussion that two branches have emerged in the interoception literature: one focuses on symbolic stimuli containing meaning and semantic information (e.g., images of emotional faces), while the other uses more ‘perceptual’ stimuli (e.g., sounds, neutral shapes, taps). The latter branch employs stimuli that do not easily elicit evocative depictions of information and are often used to test participants’ sensitivity. The disparity of results (including the current study) seems to tap into this classification. For the former, variations in the strength of external sensory data, such as facial expressions, might bias participants’ responses toward a particular interpretation, related to participants’ schemas, biases, or naturally prone responses when certain attributes of the sensorium are transiently modified.

We selected the motion discrimination task and the affect discrimination task because they represent two fundamental dimensions of visual perception: basic sensory processing and emotional interpretation. Including both tasks allowed us to investigate whether respiratory modulation affects cognitive processes across different levels. The motion discrimination task aligns with the branch of interoception research that employs perceptual stimuli (e.g., neutral shapes, sounds) to assess sensory sensitivity without

semantic content (e.g., Lacey and Lacey 1977, Johannknecht & Kayser, 2022). In contrast, the affect discrimination task corresponds to the branch focusing on affective stimuli containing meaning and emotional information (e.g., images of emotional faces). By carefully controlling the perceptual and affective content in our stimuli—a methodological strength not always present in previous studies like Zelano et al.—we aimed to minimize potential biases related to participants' schemas or inherent responses when sensory attributes are modified. This is now clarified in the opening paragraph of the results:

"We tested the effects of respiratory state on visual and affective processing using a perceptual decision-making task which alternated between each stimulus domain across blocks. These two domains were selected because they represent fundamental dimensions of visual perception - basic sensory processing and emotional interpretation - allowing us to examine whether respiratory state effects generalize across different cognitive domains. Additionally, the careful control of perceptual and affective content in our stimuli enabled us to minimize potential biases and isolate the influence of respiration on decision-making processes in both modalities."
(p. 5)

2. Along the whole paper: make clearer what is meant respiratory rhythms (brain rhythms elicited by respiration, respiratory phases of the cycle per se [as registered by the respiratory belt] or both)

We thank the reviewer for pointing us to these ambiguities. We have now clarified this nomenclature throughout the manuscript. Generally "respiratory rhythms" has been changed to "breathing" with the aim of referring to the self sustained act of lung ventilation.

3. In the intro and overall paper: clarify what is meant by modalities? (Page 5, line 78).

We have now clarified this - we now refer to one task with two visual stimulus domains throughout the manuscript.

4. Page 8, this sentences is not clear: 'To establish whether respiratory-behavioural coupling primarily depends on aligning perceptual or motor states with the respiratory cycle'

We now revise this for clarity. It now reads:

“To establish whether respiratory-behavioural coupling primarily depends on perceptual or motor systems, we analysed trials independently grouped by respiratory state at stimulus presentation (stimulus locked) and at the time of response (response locked).”

(p. 8)

Results:

A prominent finding in the literature is that subjects align their respiratory cycle with expected stimuli, improving performance. For instance, Perl et al. (2019) found participants inhaled about 1.5 seconds after task onset in various tasks, and Kluger et al. (2021) observed lower perceptual thresholds in a spatial detection task shortly after inhalation. Similarly, Grund et al. (2022) found that participants aligned their respiratory cycles with anticipated stimuli, enhancing tactile sensitivity during the transition from late inhalation to early exhalation, peaking at exhalation. Here, I truly recommend the author to check whether the participants coupled their respiration to the expected onset of the stimuli. It should not be difficult to compute and this analysis may shed more light on the current data.

We agree that this phenomenon of active sensing or utilization of respiration to improve performance is interesting. However, we intentionally wanted to avoid this to be able to sample the whole respiratory cycle: A consequence of the short trials and inter-trial intervals is, that the inter stimulus interval is generally shorter than the breaths (900 ms to 4900 ms = 66.67 to 12.25 trials per minute). Further the inter trial interval was jittered between 500 ms and 2500 ms. Both of these design features have the consequence that it is practically impossible for participants to align respiration in such a way that they receive the stimulus at a specific place in their respiration.

As illustrated in the plots below, beyond the expected effect of increased expiratory vs inspiratory cycle length, no bins of either response or stimulus onsents substantially exceed the expected values based on the numbers of samples per bin. We have now added these to the supplementary information.

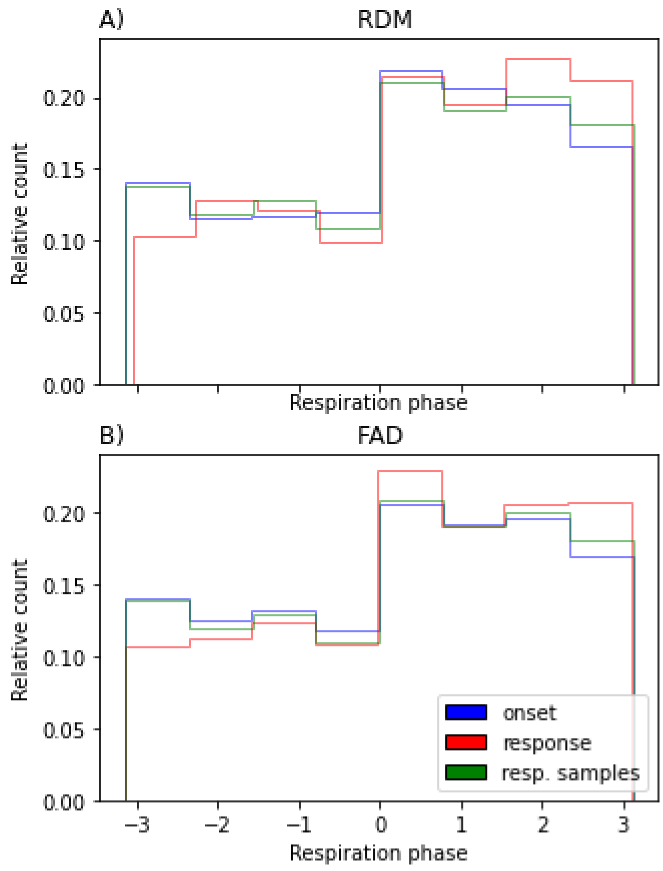

S8 Figure. Histograms of event distribution over conscientious (circular) respiratory phase. A) Random dot motion, B) Face affect discrimination. Histograms showing the relative frequency of stimulus onsets (blue lines) and responses (red lines) over the respiratory cycle. Negative pi to zero denotes the inspiratory phase and zero to positive pi denotes the expiratory phase. The green line represents the time spent in each of the phase bins. We note two things 1: As expected more time is spent at the expiratory phase compared to the inspiratory phase. 2: Relative frequency of stimulus onsets and responses follow the time spent in each phase bin.

Discussion:

There is an emerging matter in the literature that the authors could note: breathing (voluntary) as a way to control the unstoppable heart, which also introduces perceptual changes in exteroceptive tasks. The authors here could note the work of Esra al., (2020), Galvez-Pol et al., (2020, 2022), and/ or Grund et al., (2022). This could fit also the idea of respiration as an act of active sensing.

Thanks, we now include these citations in an expanded discussion.

“More generally, understanding the respiratory modulation of the brain and behavior ultimately requires a multi-modal and multi-organ perspective [See for exampel, 42–44]. In this study, we utilized computational modeling to explain how breathing influence choice, accuracy, and decision speed, extending and replicating previous findings. However, brain-body interactions encompass a complex array of physiological processes, including various neurotransmitters such as noradrenaline and autonomic signals from the heart, gut, and other visceral systems [45–48]. This complexity highlights a limitation of our current work and suggests directions for future research.“

(pp. 18-19)

Methods:

1. The rationale for using 320 near-threshold stimuli should be explained, particularly regarding how this number was determined.

Our primary goal with this design choice was to maximize the statistical power of our computational modeling within a reasonable total experiment duration. To achieve robust within-subject computational power, we needed a sufficient number of trials per participant. Pilot testing indicated that presenting 320 trials per domain allowed us to collect ample data without causing excessive fatigue or discomfort to participants. This number of trials fit well within a one-hour testing session, which we found to be the maximum duration participants could comfortably engage with the tasks while maintaining attention and performance levels. By optimizing the number of trials in this way, we aimed to enhance the signal-to-noise ratio and ensure the reliability of our findings. We have clarified our methodological approach in the revised manuscript as follows:

“After the thresholding procedure, participants completed four blocks of 80 stimuli for each domain, resulting in a total of 320 trials per domain. This number of trials was determined based on pilot studies indicating that 320 trials could be comfortably completed within a one-hour session, balancing the need for sufficient statistical power with participant comfort. In all blocks, the stimulus types were equally frequent and appeared in a pseudo-randomized order. For the RDM task, every set of 10 trials contained 5 upwards and 5 downwards motion stimuli, while for the FAD task, each set of 8 trials included 2 stimuli of each emotion level. Self-paced breaks separated the blocks to reduce fatigue. The

blocks alternated between the two domains in a counterbalanced order across participants."

(p. 22)

2. Additionally, it should be clarified whether the number of trials was sufficient to account for stimuli encoded in each respiratory phase and responded to in each phase, considering that this would ideally involve approximately 90 trials spread across inspiration and exhalation for both encoding and response phases.

We apologize for any confusion. Our analyses did not involve a 2×2 design crossing respiratory phase at both stimulus encoding and response phases. Instead, we utilized all available trials—320 per participant per task, divided between inspiratory and expiratory—for our modeling approach. This provided sufficient data to examine the main effects of respiratory state on decision-making variables. We agree that performing a full 2×2 analysis would require more trials to achieve adequate statistical power, which was beyond the scope of the current study (see also our response to R3, below). We have clarified this point in the manuscript to prevent any misunderstanding:

"It should be noted that these were analyzed separately, in order to assess the independent contributions of respiration to stimulus processing and response modulation, as the study was not designed to directly compare these conditions."

(p. 8)

3. The justification for the number of participants should be provided, based on sample size estimation and comparisons with other studies in this field.

We appreciate the reviewer's request for clarification regarding our sample size. To maximize statistical power within the practical constraints of our study, we recruited 40 participants and collected 320 trials per participant. This sample size and trial count are comparable to or exceed those of previous studies in the field. For example, Johannknecht and Kayser (2022) included 21 participants (after exclusions) with 200 trials per task; Zelano et al. (2016) had 24 participants in their second study with 180 trials; and Kluger et al. (2022) involved 30 volunteers completing 720 trials. By aligning our study with these established standards, we aimed to ensure sufficient power for our analyses and enhance the reliability of our findings. We now clarify this in a new methods text:

"To ensure adequate statistical power for our within-subject analyses, we recruited 40

participants and collected 320 trials per participant. This sample size represents a 1–3 fold increase in total statistical power compared to previous studies in this field (e.g., Johannknecht and Kayser, 2022; Kluger et al., 2022; Zelano et al., 2016), ensuring adequate power to detect and model effects typical of respiratory modulation of behavior."

(p. 21-22)

Reviewer #3:
In this study the authors designed an experiment to study the modulation of decision time in two different perceptual tasks by the respiration cycle. This study builds on previous findings on changes to both perception and response time as a function of the cardiac cycle. The study is well designed and the results are really clear. However, I have some comments that would help the reader (including me) to understand the interpretation of the results and the modelling.

Major comments.
All the significant observed effects are when the timing of the respiration cycle is taken from the response time and there were no effects when aligned to the time of stimulus onset. I might have missed this in the analysis but why was the data not analysed with an ANOVA design with factors of stimulus onset and condition? Would this not be a better test of the effects?

Thank you for your insightful comment. Our study was specifically designed to investigate whether the respiratory cycle modulates decision-making processes at either the stimulus encoding or response stage, using computational modeling to assess these effects. While we observed significant effects when aligning the respiratory cycle to the response time but not to stimulus onset, our design was not intended to test for interactions between respiratory phases at these two time points.

Conducting an ANOVA with factors of respiratory phase at stimulus onset and response would require subdividing the data into multiple conditions, significantly reducing statistical power due to fewer trials per condition. Additionally, many trials naturally share the same respiratory phase at both stimulus onset and response, complicating the independence of observations required for such an analysis. Therefore, we focused on modeling the main effects of the respiratory phase at response time, where we found the most robust effects. We have clarified this rationale in the manuscript to prevent any misunderstanding:

"It should be noted that these were analyzed separately, in order to assess the independent contributions of respiration to stimulus processing and response modulation, as the study was not designed to directly compare these conditions"
(p. 8)

Did people change the length of inspiration and expiration during the experiment? It possible to analyse the data to see if the length of the phase was modulated by HIT and MISSES trials.

To probe a possible relation between breathing behavior and trial outcomes (HITs vs. MISSES) we investigated if duration of the breath in which a trial occurred informed the outcome. For each participant we divided trials into 4 equal sized bins based on the breath duration. The resulting analyses showed no significant relation between breath duration and trial outcomes. This suggests that participants' breathing patterns remained stable and were not influenced by their performance on the tasks. We have included these analyses in the supplementary materials for enhanced clarity.

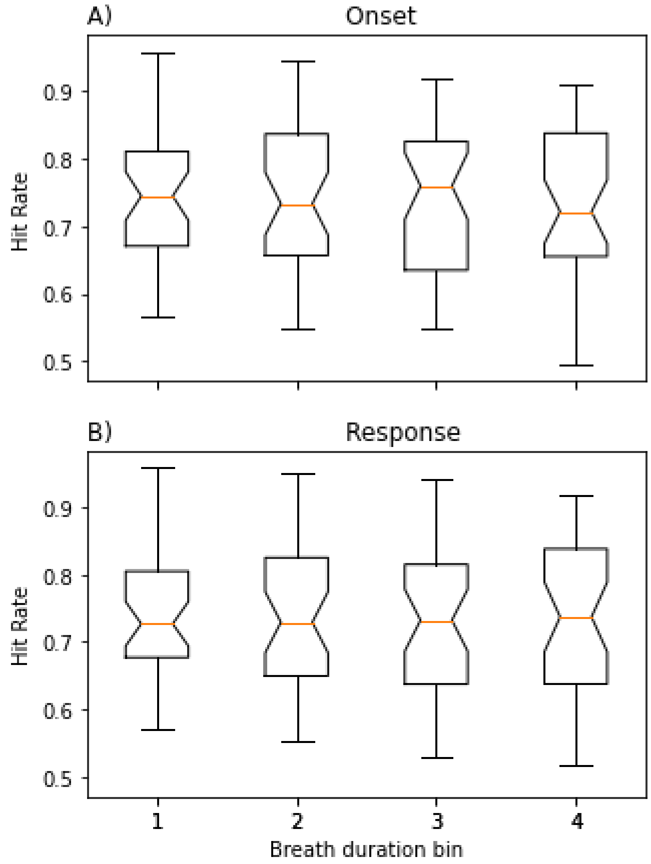

S9 Figure. Box Plots representing distribution of hit rates per breath duration quartile bin over subjects for RDM. . A) Stimulus-locked, B) Response-locked.

Relatedly - was there a difference in responses depending on when in the inspiration phase the effects occurred. Did it matter whether the stimulus or response time occurred in the first or second half of the inspiration/expiration. It was mentioned that the stimuli will go over more than one phase but it would be good to know how this actually occurred.

To address this concern, we binned the respiratory cycle into eight discrete phases and calculated the hit rates for the dot motion task and the choice rates for happy versus angry faces for each participant. We find no clear difference in hit rates according to a more fine-grained analysis of respiratory phase. We have included these analyses in the supplementary materials for enhanced clarity.

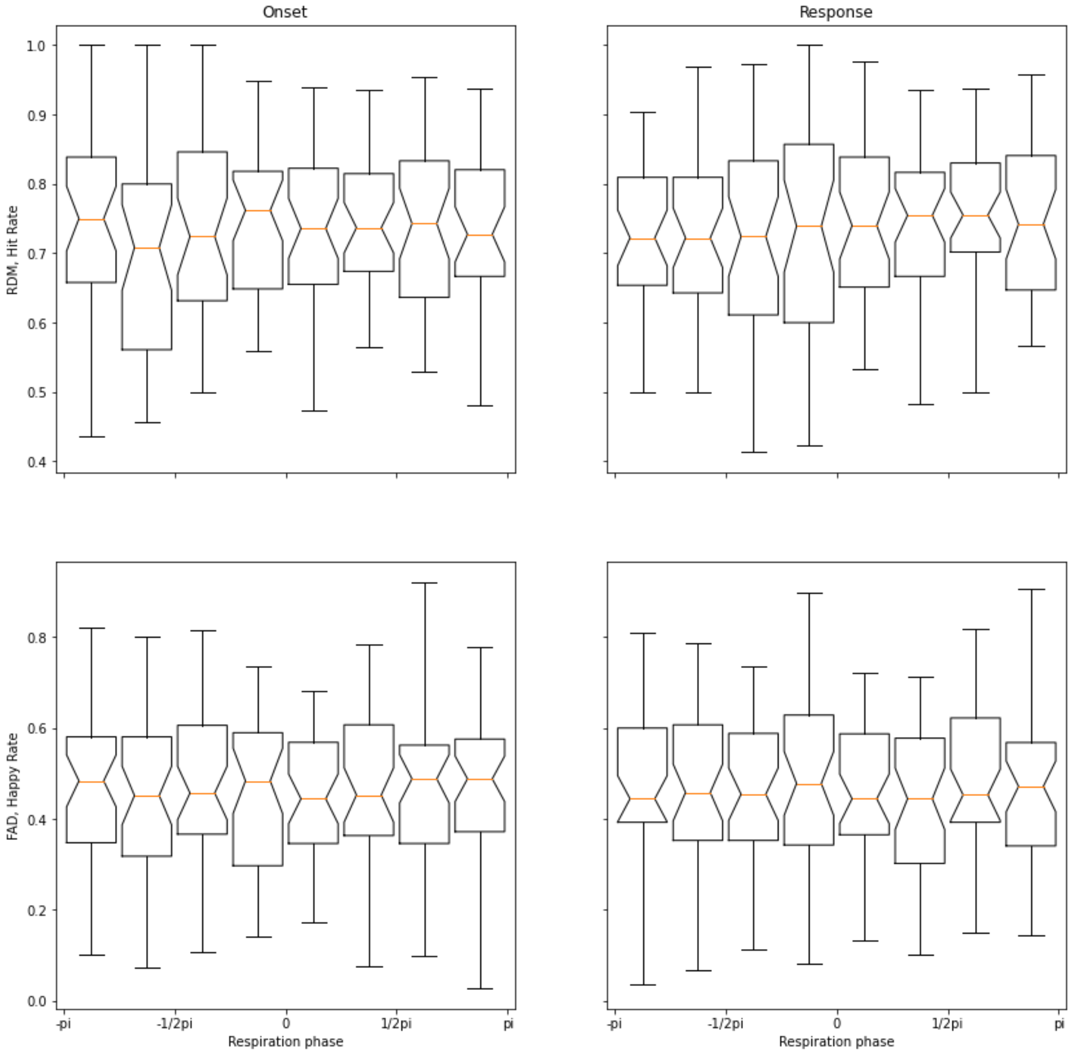

S10 Figure. Task performance over the respiratory cycle. Boxplots representing behavior in eight discrete respiratory phase bins. Stimulus- and response-locked hit rate for RDM and happy rate for FAD.

One imagines that if a subject perceived the stimulus (i.e Hits) that response time would be quicker than for a miss. Was this the case and is so does that RT effect only occur for HIT/MISSES or both. In other words does the modulation in HIT rate explain the RT change?

We confirm that response times (RTs) are generally faster for hits compared to misses, consistent with canonical findings in perceptual tasks. Misses exhibit a broader RT distribution, with both faster and slower responses more frequent than for hits. While our initial analyses treated RTs and response accuracy separately, we examined whether the modulation in hit rate explains the RT changes observed. Our additional analyses showed that the respiratory phase modulated RTs independently of correctness. Specifically, when analyzing RTs for hits and misses separately, the respiratory effects on RT remained significant within each group. See figure below for response-locked reaction time difference between inspiration and expiration for hits, misses and all trials:

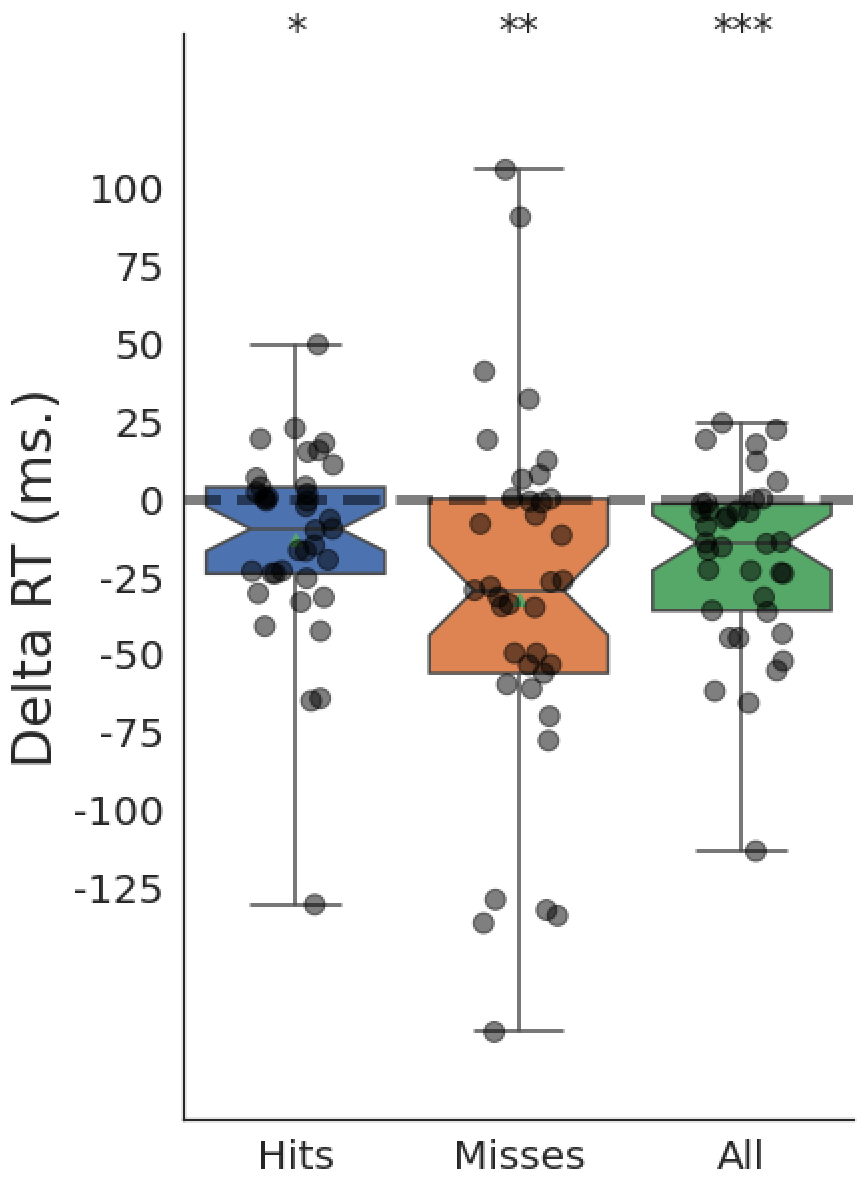

This indicates that the modulation of RT by respiratory phase is not solely due to changes in hit rate but reflects an independent influence on response timing. Therefore, respiratory modulation affects both the likelihood of correct detection and the speed of responses, suggesting distinct yet concurrent effects on perceptual decision-making and motor execution processes.

My major concern is that if there is dependency between HITs and RT (faster RT for HITS) that this would have a major impact on the modelling as the two are analysed

independently. Therefore it is really critical to show that RT is not dependent on choice for the interpretation of this data to hold.

We acknowledge your concern about the dependency between hits and reaction times (RTs) and its potential impact on our modeling. In our analysis, we employed the Drift Diffusion Model (DDM), which inherently models response accuracy (hits and misses) and RTs simultaneously. The DDM conceptualizes decision-making as a process where sensory evidence accumulates over time until a boundary is reached, resulting in both a choice and an RT. This means that the relationship between RTs and choices is not treated independently but is an integral part of the model fitting process. By fitting the DDM to both the choice data and the RT distributions, we account for the natural dependency between accuracy and RTs, ensuring that our interpretations remain valid.

Have the authors made all data and (if applicable) computational code underlying the findings in their manuscript fully available?

All study data, task code, and analysis code can be found on the project github: https://github.com/maltb/Respiratory_DDM
