# Supplementary material for: The respiratory cycle modulates distinct dynamics of affective and perceptual decision-making
Source: PLoS Comput Biol. 2025 May 27;21(5):e1013086. doi: 10.1371/journal.pcbi.1013086 (PMC12240353; doi:10.1371/journal.pcbi.1013086)
Supplement: Attachment — Submitted filename: Rebuttal_letter.pdf [file pcbi.1013086.s013.pdf]

We thank all reviewers for their comments and provide a response to their suggestion below. In response to the reviewers' suggestions, we have:

1. Updated the limitation section of the discussion

In what follows, the reviewers' comments are presented in gray, our responses in blue, changes made during the revision in red.

Reviewer #1:

Reviewer #1: I thank the authors for addressing my questions and concerns. The revised manuscript has improved and represents an interesting contribution to the emerging field of how respiration influences decision making dynamics.

In response to the comment about eye movements, the response letter from the authors states that Galvez-Pol et al show that the respiratory rhythms appear to drive saccades. I believe, the paper referring to is the 2020 publication – the aforementioned paper investigated the co-occurrence of cardiac dynamics and eye movements during visual search, and it is likely difficult to infer causality about respiration driving oculomotor responses from this work. This may be considered when the limitations about the current study are presented in the manuscript.

To address the potential of systematic eye movement effects without overstating the evidence and concern, we have updated the limitation section of the discussion. It now reads:

"Our study has a few key limitations that could be addressed in future investigations. Participants viewed stimuli which were presented at peri-threshold levels. While this manipulation ensured that respiratory effects could be observed while controlling for stimulus-specific biases, it potentially introduces confounds related to drifting participant performance over time due to cognitive fatigue. While inspection of performance across blocks of trials suggested that performance was relatively stable, future studies could utilise adaptive thresholding, which would offer the further benefit of enabling estimation of respiratory-modulated psychophysics [17].

Another important limitation is that our focus on visual perception inherently restricts the generalizability of our findings. Future research could overcome this by employing varied experimental protocols and including a broader range of sensory modalities to enhance applicability across cognitive domains. While we here focus on the commonly reported effects concerning the binary treatment of the respiratory cycle, future

studies could make use of more complex methods examining the link between continuous respiratory phase and behaviour.

Additionally, we did not collect eye tracking data. Prior work has shown that oculomotor behaviour, including microsaccades and saccades, is coupled to the cardiac cycle [42,43], which itself is modulated by respiration. It is therefore possible that our observed effects are partially mediated by breathing-induced changes in oculomotor rhythms. Future studies incorporating eye tracking will be needed to disentangle these contributions.

Finally, although our results suggest that breathing exerts distinct rhythms on visual and affective perception, comparable non-significant effects (e.g., bias in RDM and threshold in FAD; see Fig. 3) were also observed. While we utilised a relatively large number of trials and participants for a psychophysical design, these effects might have reached significance with increased power. It may be that the overall magnitude—but not the qualitative nature—of these effects is differentially modulated. Future studies using a confirmatory design (e.g., registered reports) with enhanced within- and between-subject power could help resolve this issue.

(pp. 17-18)

Reviewer #2:
Reviewer #2: The authors have correctly replied to all my questions/concerns. The manuscript was already in a very good shape, so for me it was a matter of clarifying certain points; which the authors have done. I have no further comments.
